# In silico analysis of the α-amylase family GH57: eventual subfamilies reflecting enzyme specificities

**DOI:** 10.1007/s13205-018-1325-9

**Published:** 2018-07-09

**Authors:** Mária Martinovičová, Štefan Janeček

**Affiliations:** 1 Department of Biology, Faculty of Natural Sciences, University of SS. Cyril and Methodius, Trnava, 91701 Slovakia; 2 Laboratory of Protein Evolution, Institute of Molecular Biology, Slovak Academy of Sciences, Bratislava, 84551 Slovakia

**Keywords:** α-Amylase family GH57, Bioinformatics analysis, Unique sequence/structural features, Conserved sequence regions, Evolutionary relatedness

## Abstract

Glycoside hydrolases (GHs) have been classified in the CAZy database into 153 GH families. Currently, there might be four α-amylase families: the main family GH13, the family GH57 with related GH119 and, eventually, also GH126. The family GH57 was established in 1996 as the second and smaller α-amylase family. In addition to α-amylase, it contains 4-α-glucanotransferase, α-glucan branching enzyme, amylopullulanase, dual-specificity amylopullulanase–cyclomaltodextrinase, non-specified amylase, maltogenic amylase and α-galactosidase. The family GH57 enzymes employ the retaining reaction mechanism, share five typical conserved sequence regions and possess catalytic (β/α)_7_-barrel succeeded by a four-helix bundle with the catalytic machinery consisting of catalytic nucleophile and proton donor (glutamic acid and aspartic acid at strands β4 and β7, respectively). The present bioinformatics study delivers a detailed sequence comparison of 1602 family GH57 sequences with the aim to highlight the uniqueness of each enzyme’s specificity and all eventual protein groups. This was achieved by creating the evolutionary tree focused on both the enzyme specificities and taxonomical origin. The substantial increase of numbers of sequences from recent comparisons done more than 5 years ago has allowed to refine the details of the sequence logos for the individual enzyme specificities. The study identifies a new evolutionary distinct group of α-galactosidase-related enzymes with until-now-undefined enzyme specificity but positioned on the evolutionary tree on a branch adjacent to α-galactosidases. The specificity of α-galactosidase is, moreover, the only one of the entire family GH57 for which there is no structural support for the proposal of the proton donor based on sequence analysis. The analysis also suggests a few so-called “like” protein groups related to some family GH57 enzyme specificities but lacking one or both catalytic residues.

**Electronic supplementary material:**

The online version of this article (10.1007/s13205-018-1325-9) contains supplementary material, which is available to authorized users.

## Introduction

α-Amylase (EC 3.2.1.1) is a glycoside hydrolase (GH) catalyzing in an endo-fashion the hydrolysis of α-1,4-glucosidic linkages in starch and related polysaccharides and α-glucans. Despite the fact that the catalytic action of any α-amylase should be, in principle, the same, different protein molecules may have evolved even within the same organisms to possess the same catalytic activity of the α-amylase (Janecek et al. 2014). This means that in the Carbohydrate-Active enZymes database (CAZy; http://www.cazy.org/; Cantarel et al. 2009), there have been created more α-amylase GH families reflecting especially unambiguous differences in amino acid sequences. Currently, the CAZy families GH13, GH57, GH119 and eventually also GH126 are considered as α-amylase families (Janecek et al. 2014).

The main α-amylase family, the family GH13, was established in 1991 (Henrissat 1991). At that time, regardless of the newly introduced concept of sequence-based classification of glycosidases, several α-glucan-active enzymes, e.g. cyclodextrin glucanotransferase, α-glucosidase and pullulanase, grouped around the α-amylase, were recognized to share some sequence similarities, catalytic residues and overall fold of their catalytic domain (Svensson 1988; MacGregor and Svensson 1989; Jespersen et al. 1991, 1993; Takata et al. 1992). At present, the α-amylase family GH13 represents one of the largest GH families within the CAZy database counting more than 57,000 members covering more than 30 various enzyme specificities (Janecek et al. 2014; Lombard et al. 2014). The really huge number of sequences that has still been rapidly increasing led to definition of subfamilies (Oslancova and Janecek 2002), which resulted in dividing the family into official GH13 subfamilies by CAZy curators (Stam et al. 2006), the subfamily members exhibiting a higher degree of sequence similarity to each other than to members of other GH13 subfamilies. Overall, the members of the α-amylase family GH13 employ the retaining reaction mechanism and share four–seven conserved sequence regions (CSRs; Janecek 2002), catalytic machinery and the (β/α)_8_-barrel (i.e. TIM-barrel) fold of the catalytic domain (Kuriki and Imanaka 1999; MacGregor et al. 2001; van der Maarel et al. 2002). In a wider sense, the family GH13 constitutes the clan GH-H together with related families GH70 and GH77 (Cantarel et al. 2009; Janecek and Gabrisko 2016).

The family GH57, created in 1996 (Henrissat and Bairoch 1996) has subsequently been established as the second and smaller α-amylase family (Janecek et al. 2014). In fact, it was based on the existence of sequences of two assumed α-amylases, one from thermophilic bacterium *Dictyoglomus thermophilum* (Fukusumi et al. 1988) and the other one from hyperthermophilic archaeon *Pyrococcus furiosus* (Laderman et al. 1993a), that were mutually similar, but obviously have lacked the sequence features, i.e. CSRs characteristic of the family GH13 (Janecek 1997). Although both these fundamental family GH57 members are now recognized as 4-α-glucanotransferases (Laderman et al. 1993b; Nakajima et al. 2004; Janecek et al. 2014), the family has remained to be known as the α-amylase family despite the fact that the only amylolytic enzyme characterized as the α-amylase was shown to exhibit also the pullulanase specificity (Kim et al. 2001).

The family GH57 possesses its own basic characteristics that discriminate it from the family GH13 as follows: (1) the catalytic domain adopts the fold of the so-called incomplete TIM-barrel, i.e. a seven-stranded (β/α)_7_-barrel (Imamura et al. 2003; Dickmanns et al. 2006; Palomo et al. 2011; Santos et al. 2011; Park et al. 2014; Na et al. 2017); (2) the catalytic machinery consists of two residues—a glutamic acid as a catalytic nucleophile and an aspartic acid as a proton donor located at the strands β4 and β7, respectively, of the incomplete TIM-barrel (Imamura et al. 2001; Palomo et al. 2011); and (3) there are five CSRs representing the “sequence fingerprints” of the family GH57 members (Zona et al. 2004; Blesak and Janecek 2012). Both families GH57 and GH13, however, are similar to each other in employing the same retaining reaction mechanism (Rye and Withers 2000).

With regard to families GH119 and GH126, the former containing only one experimentally confirmed α-amylase from *Bacillus circulans* (Watanabe et al. 2006) was found closely related to family GH57 (Janecek and Kuchtova 2012), whereas the latter with the only characterized amylolytic enzyme from *Clostridium perfringens* (Ficko-Blean et al. 2011) exhibiting, depending on the substrate, both endo- and exo-type of activity cannot be considered a pure α-amylase family since it exhibits also structural homology to β-glucan active endoglucanases from inverting families GH8 and GH48 (Janecek et al. 2014).

The main goal of the present study was to perform a detailed and overall bioinformatics analysis of the entire α-amylase family GH57. The study was undertaken in an effort to compare and divide the family GH57 members to as many as possible groups/subfamilies that could reflect the individual GH57 enzyme specificities and/or protein groups. The objective was to refine the “sequence fingerprints” covering the five previously described CSRs of the individual established enzyme specificities and, eventually, to identify novel, until now unrecognized GH57 groups, and thus to contribute further to the evolutionary picture of this interesting enzyme family.

## Materials and methods

### Sequence collection and comparison

Sequences were collected according to the information for the family GH57 in the CAZy database (Lombard et al. 2014), except for the specificity of maltogenic amylase (or maltose-forming amylases) that as yet has not been assigned to any CAZy family despite the fact that it was demonstrated to exhibit all the sequence-structural features characteristic of the family GH57 (Blesak and Janecek 2013; Jeon et al. 2014; Jung et al. 2014; Park et al. 2014). The sequences of maltogenic amylases, currently kept in CAZy among the “non-classified” sequences, were obtained by protein BLAST search (Altschul et al. 1990) using the sequence of maltogenic amylase from *Pyrococcus* sp. ST04 (Jung et al. 2014; UniProt accession no.: I3RE04) as a query. All studied sequences were retrieved from GenBank (Benson et al. 2018) and/or UniProt (UniProt Consortium 2017) sequence databases.

Sequences were preliminary aligned using the Clustal-X (Larkin et al. 2007) with regard to five CSRs typical for the family GH57 (Zona et al. 2004; Blesak and Janecek 2012). Those obviously lacking any of the five CSRs were eliminated from further analysis. If there was an uncertainty in identifying any of the five CSRs, a three-dimensional structure was modelled using the Phyre-2 server (http://www.sbg.bio.ic.ac.uk/phyre2/; Kelley and Sternberg 2009) and the CSR was confirmed by structure-based alignment and also structure overlay by the programme MultiProt (http://bioinfo3d.cs.tau.ac.il/MultiProt/; Shatsky et al. 2004).

The approach described above has resulted in collecting 1602 GH57 sequences (Table S1) that were divided into clusters covering previously determined enzyme specificities and those reflecting potentially novel protein groups (Table 1).

**Table 1:** Enzymes and proteins from the family GH57 used in the present study

Enzyme	Number	Archaea	Bacteria	Characterized^a^	Length^b^
α-Amylases	154	99	55	1	414
AAMY-like proteins	126	60	66		443
4-α-Glucanotransferases	107	38	69	5	670
4AGT-like proteins	63		63		623
Amylopullulanases	268	74	194	8	814
Amylopullulanases–cyclomaltodextrinases	40	20	20	4	529
APU-CMD-like proteins	5		5		728
Maltogenic amylases	34	34	–	3	590
AGAL-related enzymes	15		15		660
α-Galactosidases	14	14	–	1	362
Maltogenic amylase-like proteins	1		1		479
Non-specified amylases	170	18	152	1	785
GBE like	60		60		520
α-Glucan branching enzymes	545	26	519	4	546
Total	1602	383	1219	27	

The set was created based on sequences classified in the CAZy family GH57 completed by maltogenic amylases (currently kept in CAZy as “non-classified” sequences) using also the BLAST search

*AAMY* α-Amylase, *4AGT* 4-α-glucanotransferase, *APU-CMD* amylopullulanase–cyclomaltodextrinase, *AGAL* α-galactosidase, *GBE* α-glucan branching enzyme

^a^Characterized GH57 enzymes: α-amylase (1)—*Methanocaldococcus jannaschii* (Kim et al. 2001); 4-α-glucanotransferases (5)—*Dictyoglomus thermophilum* (Fukusumi et al. 1988; Nakajima et al. 2004), *Pyrococcus furiosus* (Laderman et al. 1993a, b), *Thermococcus kodakaraensis* (Tachibana et al. 1997), *Thermococcus litoralis* (Jeon et al. 1997; Imamura et al. 2003), *Archaeoglobus fulgidus* (Labes and Schonheit 2007; Paul et al. 2015); amylopullulanases (8)—*Pyrococcus furiosus* (Dong et al. 1997), *Thermococcus hydrothermalis* (Erra-Pujada et al. 1999), *Thermococcus litoralis* (Imamura et al. 2004), *Spirochaeta thermophila* (Angelov et al. 2010), *Dictyoglomus turgidum* (Brumm et al. 2011), *Thermococcus siculi* (Jiao et al. 2011), *Thermococcus kodakaraensis* (Guan et al. 2013), *Sulfolobus acidocaldarius* (Choi and Cha 2015); amylopullulanases–cyclomaltodextrinases (4)—*Staphylothermus marinus* (Li et al. 2013), *Caldivirga maquilingensis* (Li and Li 2015), *Desulfurococcus amylolyticus* (Park et al. 2018), *Thermophilum pendens* (Li et al. 2018); maltogenic amylases (3)—*Pyrococcus furiosus* (Comfort et al. 2008), *Pyrococcus* sp. ST04 (Jung et al. 2014; Park et al. 2014), *Thermococcus cleftensis* (Jeon et al. 2014); α-galactosidases (1)—*Pyrococcus furiosus* (van Lieshout et al. 2003); non-specified amylases (1)—uncultured bacterium (Wang et al. 2011); and α-glucan branching enzymes (4)—*Thermococcus kodakarensis* (Murakami et al. 2006; Santos et al. 2011), *Thermotoga maritima* (Ballschmiter et al. 2006; Dickmanns et al. 2006), *Thermus thermophilus* (Palomo et al. 2011), *Pyrococcus horikoshii* (Na et al. 2017)

^b^The “Length” indicates the average length

For CSRs of each identified enzyme specificity and/or protein group sequence, logos were created using the WebLogo 3.0 server (http://weblogo.threeplusone.com/; Crooks et al. 2004).

### Evolutionary analysis

The evolutionary tree of all 1602 GH57 sequences (Table 1) was calculated based on the alignment of all five CSRs as a Phylip-tree type using the neighbour-joining clustering (Saitou and Nei 1987) and the bootstrapping procedure with 1000 bootstrap trials (Felsenstein 1985) implemented in the Clustal-X package (Larkin et al. 2007). The tree was displayed with the program iTOL (http://itol.embl.de/; Letunic and Bork 2011).

### Structure comparison

Three-dimensional structure for *Thermococcus litoralis* 4-α-glucanotransferase (PDB code: 1K1Y; Imamura et al. 2003), as the family GH57 representative, was retrieved from the Protein Data Bank (PDB) (Rose et al. 2015). Three-dimensional structural models for the α-galactosidase from *Pyrococcus furiosus* (UniProt Acc. No.: Q9HHB5) and two members of the newly identified group of the α-galactosidase-related enzymes, i.e. from *Clostridium kluyveri* (UniProt Acc. No.: A5MZ16) and *Shewanella baltica* (UniProt Acc. No.: A3D6T3), were created with the Phyre-2 server (http://www.sbg.bio.ic.ac.uk/phyre2/; Kelley and Sternberg 2009). The obtained structural models were superimposed with the real structure of *T. litoralis* 4-α-glucanotransferase using the programme MultiProt (http://bioinfo3d.cs.tau.ac.il/MultiProt/; Shatsky et al. 2004) and the structures were visualized with the program WebLabViewerLite (Molecular Simulations, Inc.).

## Results and discussion

### Evolutionary relationships

The present study may represent the most complete and detailed bioinformatics analysis of the α-amylase family GH57 since it delivers a comparison of 1602 GH57 sequences (Table S1). Of these, 1568 sequences were retrieved from the family GH57 of the CAZy database directly, whereas remaining 34 sequences of the specificity of maltogenic amylase were obtained using the BLAST. This was because the three biochemically characterized maltogenic amylases have still not been classified within the family GH57, although previous in silico analysis (Blesak and Janecek 2013) along with cloning, sequencing and structural studies (Comfort et al. 2008; Jeon et al. 2014; Jung et al. 2014; Park et al. 2014) have clearly suggested they exhibit all sequence/structural features characteristic of the family GH57.

The alignment of all family GH57 proteins was originally performed using the complete amino acid sequences; however, since the sequences are too variable and, in fact, not alignable on their entire lengths (Zona et al. 2004; Blesak and Janecek 2012), further work and analysis have been based on the alignment of their five CSRs (Table S1). Since the CSRs exhibit sequence features characteristic of the individual enzyme specificities, it was reasonable to group the sequence newly collected in the present study with the already recognized enzyme specificities of the family GH57.

**Fig. 1:**
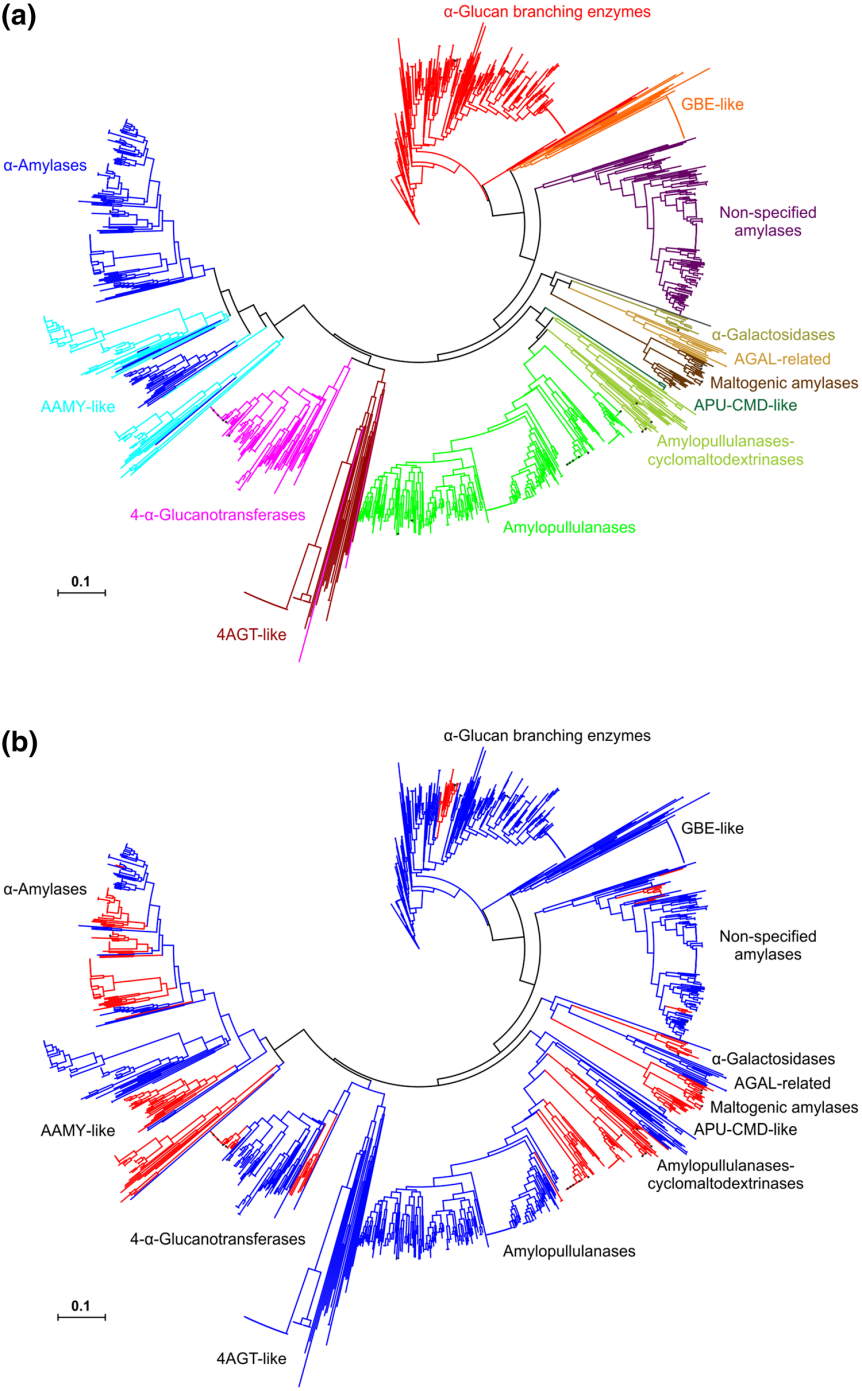
Evolutionary tree of the α-amylase family GH57. The analyzed set contains 1602 sequences (for details, see Table 1). The tree is based on the alignment of conserved sequence regions (cf. Fig. 2) and calculated using the neighbor-joining method. The picture shows the same tree emphasizing **a** its division according to individual enzyme specificities and protein groups and **b** the division reflecting the taxonomic origin—bacteria (blue) and archaea (red). The 27 experimentally characterized GH57 enzymes (cf. Table 1) are signified by black dots in both versions of the tree. *GBE* α-glucan branching enzyme, *AGAL* α-galactosidase, *APU-CMD* amylopullulanase–cyclomaltodextrinase, *4AGT* 4-α-glucanotransferase, *AAMY* α-amylase

The overall division of all 1602 sequences from the studied set into the individual enzyme specificities and/or protein groups is illustrated by the evolutionary tree (Fig. 1a). The tree contains clusters of family GH57 enzymes, such as α-amylase, 4-α-glucanotransferase, amylopullulanase, bifunctional amylopullulanase–cyclomaltodextrinase, maltogenic amylase, α-galactosidase, non-specified amylase and α-glucan branching enzyme, well established by previous studies (Blesak and Janecek 2012, 2013). With regard to their mutual evolutionary relationships, α-amylase are clustered together with 4-α-glucanotransferase; both being in a closer relatedness with amylopullulanases and their bifunctional counterparts possessing also the cyclomaltodextrinase specificity. Next to them, there is cluster covering closely related maltogenic amylases with α-galactosidases, containing interestingly also a potential newly discovered group α-galactosidase-related enzymes. This group may eventually represent even a new GH57 specificity because the sequences of its members possess a complete family GH57 catalytic machinery. In the remaining part of the evolutionary tree, at the site opposite to α-amylases and 4-α-glucanotransferases, there are clusters of the non-specified amylases and α-glucan branching enzymes.

In addition, there are several groups of hypothetical GH57 proteins, which are closely related to a given enzyme specificity, but they lack one or even both catalytic residues. These are very probably not able to play the role of a typical family GH57 enzyme. Such a special group with incomplete catalytic machinery was first observed for the specificity of α-amylase and was named as the group of α-amylase-like proteins (Janecek and Blesak 2011). This study brings the analogous groups of “like” proteins for a few additional enzyme specificities, i.e. 4-α-glucanotransferase, amylopullulanase-cyclomaltodextrinase and α-glucan branching enzyme (Fig. 1a). It should be pointed out that the groups of the “like” proteins are not in all four cases absolutely homogeneous, i.e. there are some exceptions that contain the complete catalytic machinery, e.g., not only mainly among the α-amylase-like proteins but also, although rather rarely, among the “like” proteins related to 4-α-glucanotransferases and α-glucan branching enzymes. Note that there is only one GH57 sequence (originating from *Opitutaceae bacterium*; Fig. 1a) that—due to sequence differences even within the five CSRs—has been positioned on a separate branch and thus has not been classified with any of the above-mentioned family GH57 groups.

The evolutionary tree shown in Fig. 1b illustrates the division of the individual sequences with regard to taxonomy, i.e. their either bacterial or archaeal origin. It is evident that overall *Bacteria* dominates over the *Archaea* within the family GH57 (Lombard et al. 2014). It is of interest that some enzyme specificities like that of the α-glucan branching enzymes are almost completely of bacterial origin although, on the other hand, e.g. all maltogenic amylases originate only from archaeons (Fig. 1b). Interestingly, all the sequences from the groups of α-glucan branching enzyme-like, 4-α-glucanotransferase-like and amylopullulanase–cyclomaltodextrinase-like proteins are represented by bacterial producers only. The α-galactosidases and α-galactosidase-related enzymes occupying the two adjacent branches of the tree are of archaeal and bacterial origins, respectively (Fig. 1b).

### Sequence logos

**Fig. 2:**
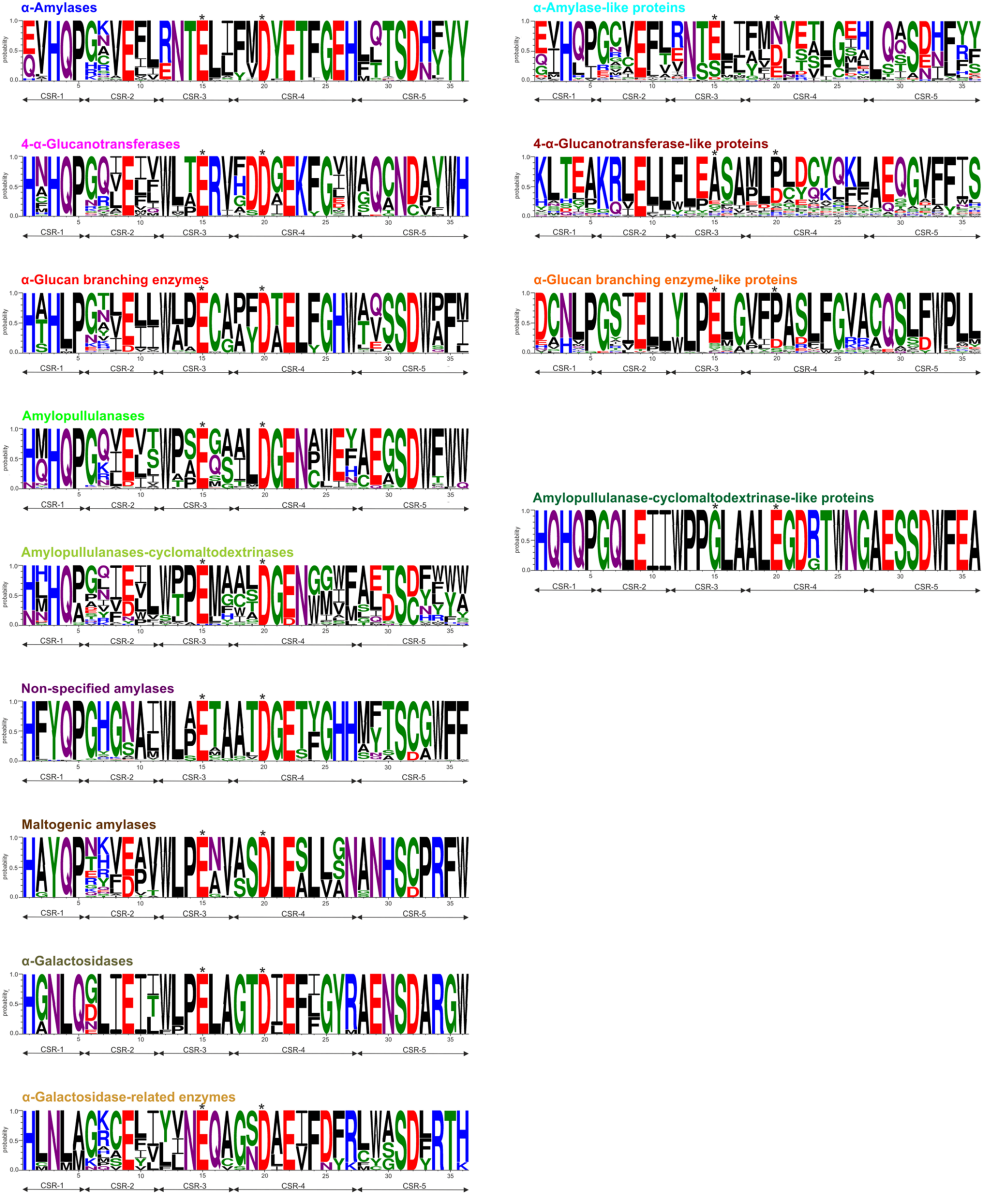
Sequence fingerprints of GH57 enzyme specificities and their “like” protein homologues. Sequence logos of enzyme specificities (on the left) are based on 154 α-amylases, 268 amylopullulanases, 40 amylopullulanase–cyclomaltodextrinases, 107 4-α-glucanotransferases, 545 α-glucan branching enzymes, 34 maltogenic amylases, 170 non-specified amylases, 14 α-galactosidases and 15 α-galactosidase-related enzymes. Sequence logos of their “like” protein homologues (on the right) are based on 126 sequences of α-amylase-like proteins, five sequences of amylopullulanase–cyclomaltodextrinase-like proteins, 63 sequences of 4-α-glucanotransferase-like proteins, 60 sequences of α-glucan branching enzyme-like proteins and one sequence of maltogenic amylase-like protein. *CSR-1* residues 1–5, *CSR-2* residues 6–11, *CSR-3* residues 12–17, *CSR-4* residues 18–27, *CSR-5* residues 28–36. The catalytic nucleophile (No. 15, Glu) with the proton donor (No. 20, Asp) and their corresponding positions in the “like” protein counterparts are indicated by asterisks

The importance of the five CSRs defined first by Zona et al. (2004) has already been demonstrated using their sequence logos (Blesak and Janecek 2012, 2013) that can be used as the so-called sequence fingerprints of the individual enzyme specificities. This is due to the fact that all five CSRs (Fig. 2) represent the best conserved segments of the family GH57 members, there are also some unique positions within the logos attributable to a given enzyme specificity only discriminating that specificity from remaining ones (Janecek et al. 2014). This study delivers thus a revisited view of the five family GH57 CSRs because the previous in silico studies (Janecek and Blesak 2011; Blesak and Janecek 2012, 2013) mapped the situation more than 5 years ago (2011–2013) with the total number of compared sequences reaching 554 sequences in comparison with 1602 sequences studied here identifying also some novel enzymatic and/or protein groups (Fig. 1). The approximately 300% increase in the number of sequences has, however, resulted in the fact that some sequence features described in original studies (Blesak and Janecek 2012, 2013) as absolutely unique for some specificities have not necessarily kept their total uniqueness until now.

**Fig. 3:**
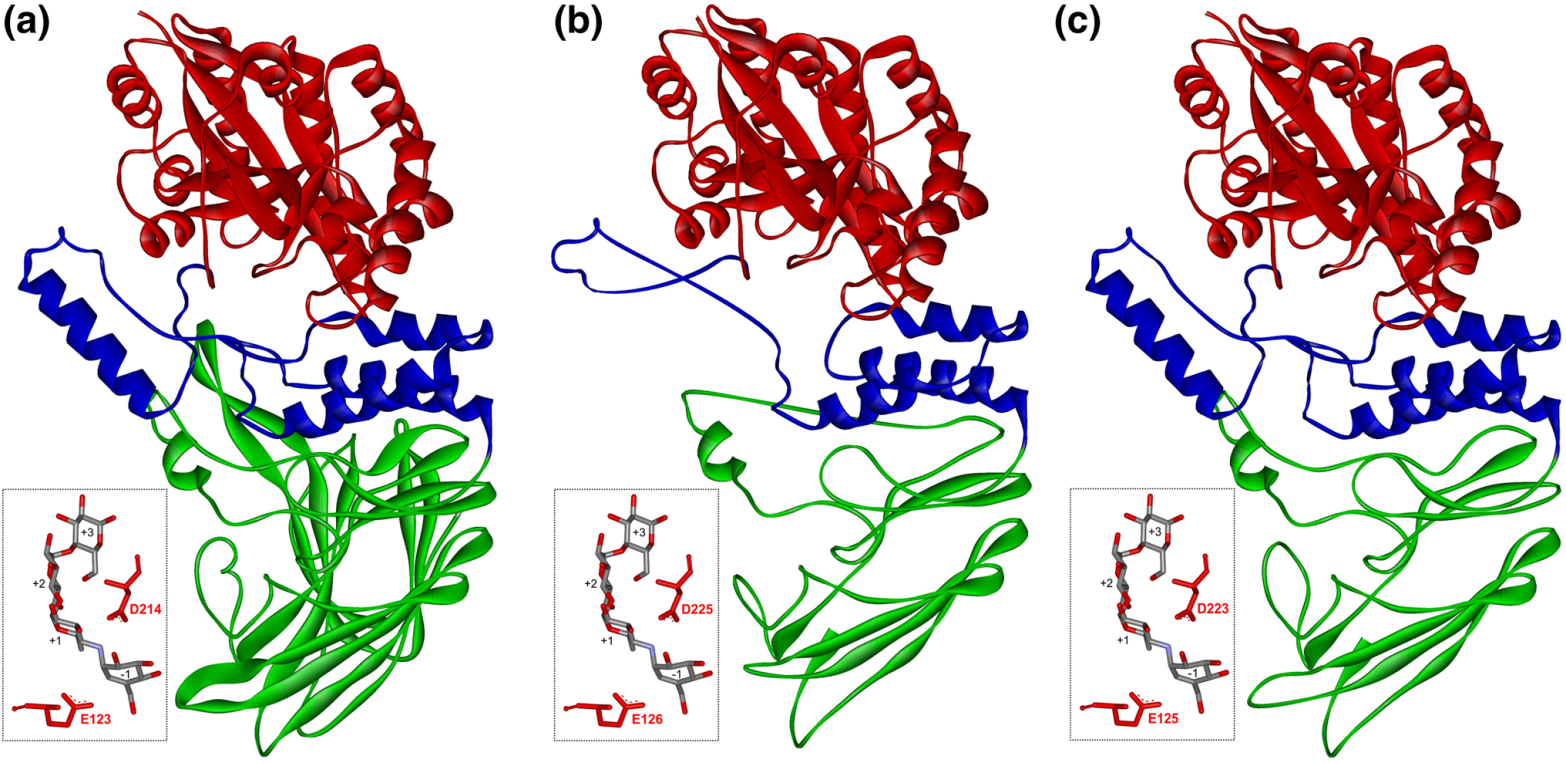
Structure comparison of 4-α-glucanotransferase and α-galactosidase-related enzymes. **a** Real structure of *Thermococcus litoralis* 4-α-glucanotransferase (Imamura et al. 2003; PDB: 1K1Y); **b** and **c** modelled structures of α-galactosidase-related enzymes from *Shewanella baltica* and *Clostridium kluyveri*. The individual domains are coloured as follows: catalytic incomplete TIM-barrel—red, succeeding helical bundle—blue, and C-terminal β-sheet domain—green. Detailed view illustrates the active site with glutamic acid and aspartic acid as catalytic nucleophile and proton donor, respectively, with bound acarbose (from the 4-α-glucanotransferase structure)

For sequences of α-amylases, positions 1 (CSR-1) and 12 (CSR-3) occupied mostly by a glutamic acid (or glutamine) and arginine (or glutamic acid) can be considered as important ones, but the most important sequence feature of the logo has been identified at its end in positions 35 and 36 with two tyrosines in the case of α-amylases. With regard to position 12 (beginning of the CSR-3), there is almost exclusively found a tryptophan (or at least an aromatic residue) in all specificities except for the α-amylase and α-amylase-like proteins. The two last positions of the logo represent, on the other hand, the best sequence feature distinguishing the individual family GH57 enzyme specificities from each other (Fig. 2). For example, the non-specified amylases possess there two phenylalanines, maltogenic amylases have phenylalanine succeeded by tryptophan, amylopullulanases have two tryptophans (although not unambiguously), whereas α-glucan branching enzymes possess there just a phenylalanine succeeded by a non-invariantly conserved non-aromatic residue and α-galactosidases contain only the tryptophan preceded by an invariant glycine (Fig. 2). As far as the newly identified group of α-galactosidase-related enzymes is concerned, this seems to have no one of the two positions (35 and 36) occupied by any aromatic residue; only threonine and lysine (histidine) can be found there (Fig. 3).

Despite the slight modifications within some positions proposed previously as exclusive features of the individual enzyme specificities (Janecek and Blesak 2011; Blesak and Janecek 2012, 2013), it is still obvious that, in addition to the end of the logo described above (positions 35 and 36), the position 23 defined by Blesak and Janecek (2012) should still be seriously taken into account. For most of specificities, there is always an almost invariantly conserved but different residue, such as threonine/serine/alanine for α-amylases/non-specified amylases/maltogenic amylases, lysine for 4-α-glucanotransferases, leucine for α-glucan branching enzymes and phenylalanine for α-galactosidases and α-galactosidase-related enzymes (Fig. 2). The position occupied by a cysteine residue proposed previously as unique for α-glucan branching enzymes (Blesak and Janecek 2012) has also lost its total invariance since in a few cases the cysteine is replaced by a methionine, like in the case of the α-glucan branching enzyme from *Thermus thermophilus* (Palomo et al. 2011).

With regard to the groups of family GH57 proteins named as the “like” proteins, their sequence logos also exhibit their own characteristic features, the most prominent one being the substitution of one or both catalytic residues, as originally defined by Janecek and Blesak (2011). Their detailed in silico analysis will be, however, described elsewhere.

### Tertiary structure comparison

Since the three-dimensional structures of the family GH57 enzymes that can be considered as being “amylolytic” ones have already either been experimentally determined (4-α-glucanotransferase, α-glucan-branching enzyme and maltogenic amylase) or modelled (α-amylase, non-specified amylase and amylopullulanase), the structural analysis here has been focused on the group of less deeply studied α-galactosidases and on the new group found on the adjacent branch of the evolutionary tree, the so-called group of α-galactosidase-related enzymes (Fig. 1). The three-dimensional structure models were prepared by the homology modelling for the biochemically characterized α-galactosidase from *Pyrococcus furiosus* (van Lieshout et al. 2003) and two hypothetical α-galactosidase-related enzymes (differing between each other in their length) from *Shewanella baltica* (640 residues; a typical length for this group) and *Clostridium kluyveri* (747 residues).

It should be pointed out, however, that for the α-galactosidase neither the homology modelling using the Phyre-2 server (Kelley and Sternberg 2009) nor the structural comparison using the MultiProt server (Shatsky et al. 2004) has supported the sequence comparison completely with regard to identification of both catalytic residues. While the catalytic nucleophile, i.e. Glu117 in the *Pyrococcus furiosus* α-galactosidase, has perfectly matched with the corresponding glutamic acid counterpart from all real template structures of α-glucan branching enzymes and 4-α-glucanotransferase (Imamura et al. 2003; Dickmanns et al. 2006; Palomo et al. 2011; Santos et al. 2011; Na et al. 2017), the catalytic proton donor, i.e. Asp248 suggested within the frame of the CSR-4 (Fig. 2), has not overlapped with the required aspartic acid residue from any of structural templates mentioned above. Note that the proton donor was found neither in the only experimental study dealing with α-galactosidases (van Lieshout et al. 2003). It is thus reasonable to suggest that to solve this issue satisfactorily, more experimental work is still necessary, e.g., preparing and characterizing the site-directed mutants of the Asp248 in the α-galactosidase from *Pyrococcus furiosus* and of other candidate positions, in addition to crystallography trials.

With regard to the two α-galactosidase-related enzymes, their homology models have unambiguously confirmed the predicted catalytic machinery from sequence logos (Fig. 2), i.e. catalytic glutamic acid nucleophile in the CSR-3 and proton donor aspartic acid in the CSR-4. The models have spanned the regions 5-516 of 640 and 1-613 of 747 residues, respectively, in *Shewanella baltica* and *Clostridium kluyveri* α-galactosidase-related enzymes. Of the models, 481 and 541 residues were superimposed with the template real structure of the 4-α-glucanotransferase from *Thermococcus litoralis* (Imamura et al. 2003) in the former and latter enzymes with the zero Å root mean-square deviation value in both cases. The potential catalytic machinery for α-galactosidase-related enzymes from *Shewanella baltica* and *Clostridium kluyveri* should thus consist of Glu126 and Asp225 and of Glu125 and Asp223, respectively.

## Electronic supplementary material

Below is the link to the electronic supplementary material.

Supplementary material 1 (XLSX 177 KB)

## Abbreviations

CSR: Conserved sequence region
GH: Glycoside hydrolase
PDB: Protein Data Bank
